# Interactive Psychometrics for Autism With the Human Dynamic Clamp: Interpersonal Synchrony From Sensorimotor to Sociocognitive Domains

**DOI:** 10.3389/fpsyt.2020.510366

**Published:** 2020-11-26

**Authors:** Florence Baillin, Aline Lefebvre, Amandine Pedoux, Yann Beauxis, Denis A. Engemann, Anna Maruani, Frédérique Amsellem, J. A. Scott Kelso, Thomas Bourgeron, Richard Delorme, Guillaume Dumas

**Affiliations:** ^1^Human Genetics and Cognitive Functions, Institut Pasteur, UMR3571 CNRS, Université de Paris, Paris, France; ^2^Child and Adolescent Psychiatry Department, Robert Debré Hospital, Paris, France; ^3^Parietal Project-Team, INRIA Saclay – Île de France, Palaiseau, France; ^4^Human Brain and Behavior Laboratory, Center for Complex Systems and Brain Sciences, Florida Atlantic University, Boca Raton, FL, United States; ^5^Intelligent Systems Research Centre, University of Ulster, Derry Londonderry, United Kingdom; ^6^Department of Psychiatry, Université de Montréal, Montreal, QC, Canada; ^7^CHU Sainte-Justine Centre de Recherche, Precision Psychiatry and Social Physiology Laboratory, Montreal, QC, Canada

**Keywords:** computational psychiatry, human-machine interface (HMI), psychometric, interpersonal synchrony, autism spectrum disorder, coordination dynamics

## Abstract

The human dynamic clamp (HDC) is a human–machine interface designed on the basis of coordination dynamics for studying realistic social interaction under controlled and reproducible conditions. Here, we propose to probe the validity of the HDC as a psychometric instrument for quantifying social abilities in children with autism spectrum disorder (ASD) and neurotypical development. To study interpersonal synchrony with the HDC, we derived five standardized scores following a gradient from sensorimotor and motor to higher sociocognitive skills in a sample of 155 individuals (113 participants with ASD, 42 typically developing participants; aged 5 to 25 years; IQ > 70). Regression analyses were performed using normative modeling on global scores according to four subconditions (HDC behavior “cooperative/competitive,” human task “in-phase/anti-phase,” diagnosis, and age at inclusion). Children with ASD had lower scores than controls for motor skills. HDC motor coordination scores were the best candidates for stratification and diagnostic biomarkers according to exploratory analyses of hierarchical clustering and multivariate classification. Independently of phenotype, sociocognitive skills increased with developmental age while being affected by the ongoing task and HDC behavior. Weaker performance in ASD for motor skills suggests the convergent validity of the HDC for evaluating social interaction. Results provided additional evidence of a relationship between sensorimotor and sociocognitive skills. HDC may also be used as a marker of maturation of sociocognitive skills during real-time social interaction. Through its standardized and objective evaluation, the HDC not only represents a valid paradigm for the study of interpersonal synchrony but also offers a promising, clinically relevant psychometric instrument for the evaluation and stratification of sociomotor dysfunctions.

## Introduction

Autism spectrum disorder (ASD) is a complex neurodevelopmental disorder (1) defined by the co-occurrence of social communication problems, repetitive behaviors, and restricted interests. The prevalence of ASD has increased in recent years from <1 in 1,000 individuals to 1 in 58 (2, 3). With different levels of severity of symptoms, ASD is highly heterogeneous, both phenotypically (4) and genetically (5). More than 50% of patients suffer from at least four other psychiatric comorbid conditions (6). This strong heterogeneity complicates the development of psychometric assessment tools that allow for a personalized and thorough evaluation of a child's skills (7). Identification of robust, valid, and quantitative biomarkers of social communication disability, a key symptom of ASD, is thus a major societal challenge for improving early diagnosis and individualized care.

As a keystone of social communication, interpersonal synchrony (IS) is a fundamental aspect to explore in order to better understand and apprehend ASD. IS can be defined as a rhythmic matching of actions in time and in phase with another person based on nonverbal behaviors (8). IS comprises multiple components, involving sociocognitive, sensory motor, and motor skills, as well as adaptive capacities (9, 10). At the behavioral level, IS can be measured through microlevel detection of bonding-related behaviors (11), frame-by-frame analysis of video (12), or even using machine learning tools (13).

In this context, the human dynamic clamp (HDC) is a new paradigm of human–machine interaction based on the science of coordination (coordination dynamics) that enables the study of the neurobehavioral processes involved in IS (14–16). Controlled using empirically grounded models of coordination dynamics (17), the HDC allows a dynamic bidirectional interaction in real time between a human and a virtual avatar. The HDC paradigm has already been validated empirically in adults (15, 18, 19). Using high-resolution electroencephalography, it recently revealed how distributed neural dynamics integrate information from “low-level” sensorimotor mechanisms and “high-level” sociocognitive processes such as intention attribution or judgment of humanness (18). Using skin potential responses, we demonstrated that HDC is able to induce emotional reaction, especially when human participants believed that their partner was human and when movement coordination was stable (19). Finally, we also introduced the virtual teacher (VT) configuration that allows human participants to change their behavioral repertoire by internalizing new interpersonal coordination patterns (e.g., nontrivial relative phase between movements of the two interacting partners), thereby opening possibilities of applying HDC to rehabilitation (15).

IS seems to be substantially impaired in children and adolescents with ASD (20, 21). A few studies among children (6–11 years old) (22) and adolescents (10–16.5 years old) (23) have explored IS in automated motion analysis to quantify movements of body parts. Still, the exploratory paradigms are mainly rhythmic in children with ASD (3.5–10 years old) (24–27) and in adolescents (12–17 years old) (28, 29). However, even if children with ASD face difficulties in movement coordination during a social exchange, social embodiment seems preserved and appears to correlate with social cognitive ability (22).

One hypothesis currently under investigation suggests that motor and sensory motor skill development are linked to social cognition and cognitive development (25, 30). ASD is frequently found to be associated with difficulties in attributing mental states to oneself and to others (31), where intention attribution is characterized by an appraisal based on the intention underlying someone else's action (32). In addition to primary dysfunctions in social communication skills, deficits in perceptual–motor performance are found in between 50 and 80% of children diagnosed with ASD (moving with awareness, integrated self, proprioceptive feedback, visuo-perceptual performance, sensory integration) (23, 25, 33–37). About 80% also show motor skill impairments such as praxis, basic motor control, postural control, gait abnormalities, motor coordination, manual dexterity, gross and fine motor skills, and gestures in complex movement sequences (20, 25, 38).

Interventions targeting the development of IS are promising and show evidence for plasticity (39–41). Early detection and intervention directly focusing on the development of IS showed preliminary evidence of positive effects on motor and communication skills (42), especially later in both language and social abilities (39). Such evidence supports IS as a potent tool for the diagnosis and care of ASD children.

Up to now, language, cognitive ability, social engagement, and motor skills have emerged as the most robust predictors of ASD among toddlers (43–45) and during childhood and adolescence (46). Thus, early dysfunction in IS could have cascading consequences and even participate in explaining the heterogeneity of ASD. Such observations reflect the difficulty of assessment by means of reliable and age-scalable markers of IS and the need for personalized analysis [as has been done, for example, in studies of skill learning, cf. (47)].

In the present work, we first validate how the HDC measures different behavioral processes involved in social dynamic interactions in children with neurotypical development, and then evaluate how the HDC can assess IS alterations in children with ASD. A secondary objective is to standardize the test and develop indicators that measure and identify sociocognitive and sensorimotor markers. In order to highlight the specific heterogeneity of ASD compared to typical neurodevelopment, developmental trajectories are integrated into our analysis using normative modeling (48), and HDC behavioral measures are tested as reproducible and reliable clinical markers.

## Methods

### Sample

We enrolled in the study a sample of 156 individuals composed of 114 participants with ASD and 42 participants with typical development (Table 1). All participants were recruited at the Child Psychiatry Department of the Robert Debré University Hospital, Paris (France).

**Table 1:** Demographic and clinical characteristics of the participants enrolled in the study.

	**Children with ASD (*n* = 113)**	**Children with typical development (*n* = 42)**	**Group test; *p* value**
Gender (m/f)	96/18	25/17	χ^2^ = 9.37; *p* = 0.002
Age at inclusion	11.2 ± 3.2	16 ± 4.4	*t* = −7.51; *p* = 4.6e-12
SRS *t* score	74.2 ± 12	45 ± 5.4	*t* = 14.20; *p* = 1.5e-28
Full-scale intellectual quotient	101.2 ± 18.5	107.4 ± 13.2	*t* = −1.88; *p* = 0.06
Right handedness	92/21	38/11	χ^2^ = 1.47; *p* = 0.23

*Mean values and respective standard deviations for continuous variables. n, sample size; ASD, autism spectrum disorder; IQ, intellectual quotient; SRS t score, Social Responsiveness Scale t score*.

Patients with ASD were included after a systematic clinical and medical examination, including negative blood test results for Fragile-X and the exclusion of participants carrying a large deletion over 2 Mb as detected by the Illumina 700 SNPs array. The final diagnosis of ASD was based on DSM-5 criteria and outcomes from the Autism Diagnostic Observation Schedule-Second Edition (ADOS-II) (49), the Autism Diagnostic Interview-Revised (ADI-R) (50), and the Social Responsiveness Scale−2nd edition (SRS-2) (51) for the dimensional diagnosis of social skills and data from experts in the field. Intellectual functioning for all participants was estimated using the Wechsler Intelligence Scale for Children and Adolescents−5th edition (WISC-V) (52). The current threshold for intellectual disability (i.e., IQ <70) was used, following international standards (DSM-5). Among participants, 14 children (controls = 2, ASD = 12) were below 85 and 33 (controls = 14, ASD = 19) were above 115. Participants with normal neurotypical development were from the general population and reported no personal or familial history of ASD or axis I psychiatric conditions requiring specific needs.

An assessment of dexterity and motor coordination of hands and fingers was made using the Purdue Pegboard (53). For the present study, we used the versions with charts defined on a population aged 5 to 15 years 11 months and beyond the age of 16 years (54, 55). Only the “preferred hand score,” viz. place the most items using the preferred hand in a row in 30 s, was conserved. A *z* score was calculated according to age and gender. Children were also assessed with the Child Neuropsychological Assessment—second edition (NEPSY-II) (56) to specifically explore affect recognition (AF) and theory of mind (TOM).

The research was carried out in accordance with the recommendations of the local ethics committee of Hospital Robert Debré. All the parents of participants gave written informed consent in accordance with the Declaration of Helsinki. The protocol was approved by the INSERM Ethics Committee (study approval no. 08-029).

### The Human Dynamic Clamp Paradigm

**Figure 1:**
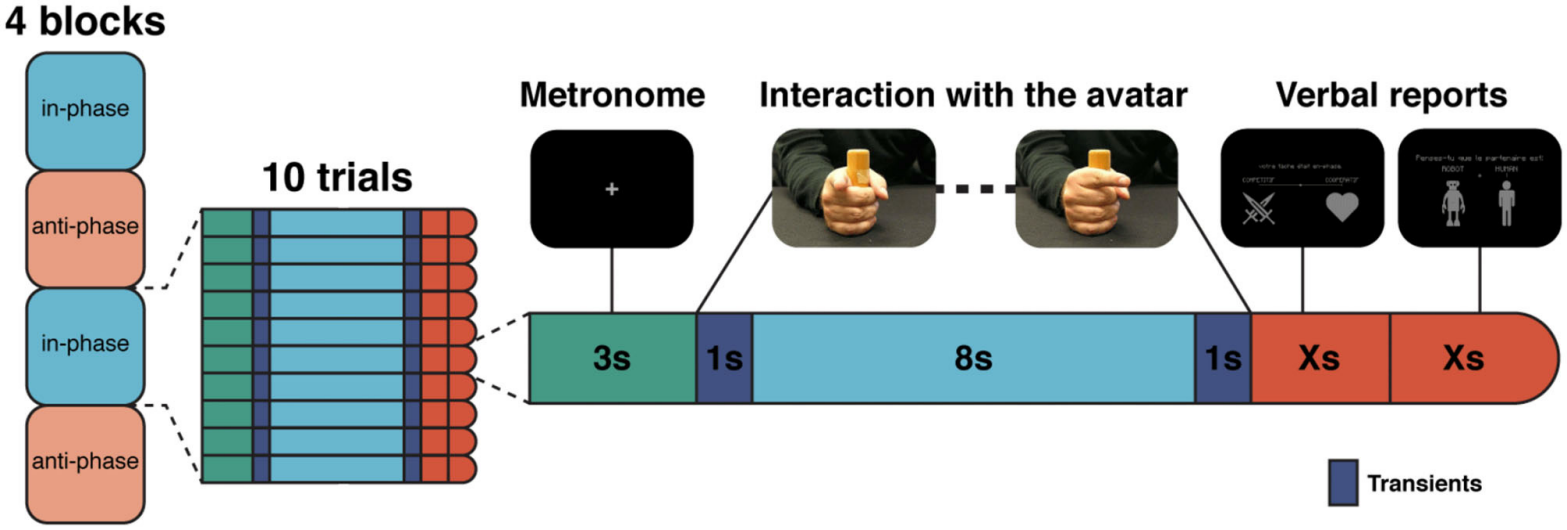
Experimental design. Structure of the protocol with four blocks alternating “in-phase/anti-phase.” Each block is divided into 10 trials (left). Each trial starts with the participant instructed to synchronize with the sound of a metronome for 3 s. Then, the participant interacts with the avatar according to the instruction (e.g., “in-phase”). At the end of the trial, two questions appear directly on the screen. First, the impression of the participant on the competitive or cooperative behavior of the avatar, and second regarding the humanness of the avatar (right). Shown are screenshots of what participants could see (top). In-phase refers to synchronized movements using homologous muscles of the limbs and anti-phase to alternating movements (180° out of phase). Cooperative and competitive refer respectively to the behavior of the virtual partner when it has shared or conflicting goals with the human participant.

The HDC system (14–16) is a human–machine interface consisting of three parts: (1) a sensor measuring the movement of the participant's index finger which is fed into (2) a mathematical model integrating the position and velocity of the human's movement to simulate in real time [*via* the HKB model; (57)] the behavior of a virtual partner or avatar and 3) a screen facing the participant where the resulting finger movements of the virtual partner (VP) appear as a human hand. The HDC software computes in real time the corresponding position of the VP (Figure 1). At the beginning of each trial, an instruction was given to the participant to synchronize her/his movement in-phase (i.e., synchronize her/his movements to those of the VP) or anti-phase (i.e., synchronize her/his movements with a half-period offset with the VP's phase). In this experiment, while the partner is a virtual partner throughout, all participants were instructed that half the time the partner is virtual (i.e., movements are computer-driven) and half the time the partner is a real sex- and age-matched human performing the same task in another room of the hospital. The protocol was composed of 40 trials, divided into four blocks. The instructions to the participant stayed the same within each block. The instruction for the first block was randomly assigned at the beginning of the experiment. During the trials, the VP could adopt a “cooperative” or “competitive” behavior, meaning that it shares the same goal or the opposite goal to the one assigned to the participant (i.e., VP aims to move its finger in in-phase coordination when the participant aims to move his finger in anti-phase and vice versa, exactly as if the partner was not cooperating with but in opposition to the participant). Behavior of the VP was randomized across all trials, disregarding block structure. At the end of each trial, the participant was asked if s/he felt like s/he was playing with a human or a VP and to quantify the level of cooperativeness or competitiveness of the partner (see also 88).

### HDC Behavioral Measures

In the present study, five normalized scores (between 0 and 1, 0 being the worst) of the HDC paradigm were automatically aimed at evaluating dimensions of social cognition, ranging from sensory motor to representational aspects: (1) a motor score which measures the difference of amplitude of imitative gestures between the participant and the VP; (2) a coordination score which corresponds to the temporal index of imitation; (3) a task score which is based on how well the ongoing relative phase of the VP and the participant match, taking into account the task condition; (4) an intention score which evaluates the ability of the participant to properly attribute intention toward the “cooperative” or “competitive” behavior of the VP; and (5) a humanness score which reflects quantitatively the impression of the participant on the human or robotic character of the partner (see Supplementary Material for more details).

### Data Analysis Using Normative Modeling

All statistical data analyses were performed using Python 3.7 (58) [numpy 1.17.2 (59, 60) and scipy 1.3.1 (61)]. Normative modeling (NM) provides a metric similar to a *z* score, but accounts for the underlying structure of the population across multiple covariates. NM uses Gaussian processes (GP) to model the distribution of control group measures while estimating separately the overall trajectory in the covariate space, the heterogeneity in the population, and the uncertainty of the fit (62). The Python code is available in open access at https://github.com/GHFC/SoNeTAA/.

## Results

### Sociodemographic and Group Comparative Analyses

Overall, participants with ASD were younger than the control group [*t*(154) = 2.6, *p* = 2.6e-11], with a larger male/female ratio (Fisher exact, *p* = 0.002) than the control group. No statistically significant differences were found for IQ and handedness. As expected, the group with ASD scored higher in the SRS [*t*(154) = 14.3, *p* = 7.4e-29]. No statistically significant differences were found for IQ and handedness. The group with ASD scored lower on all the standardized psychometric instruments assessing social skills: NEPSY-II TOM total score (Mann–Whitney *U* = 104.5, *p* = 0.0005), NEPSY-II AF raw (*U* = 114, *p* = 0.0017), and the Purdue Pegboard, the validated task assessing motor coordination skills (*U* = 138, *p* = 0.0006).

### Developmental Trajectories of HDC Scores

**Figure 2:**
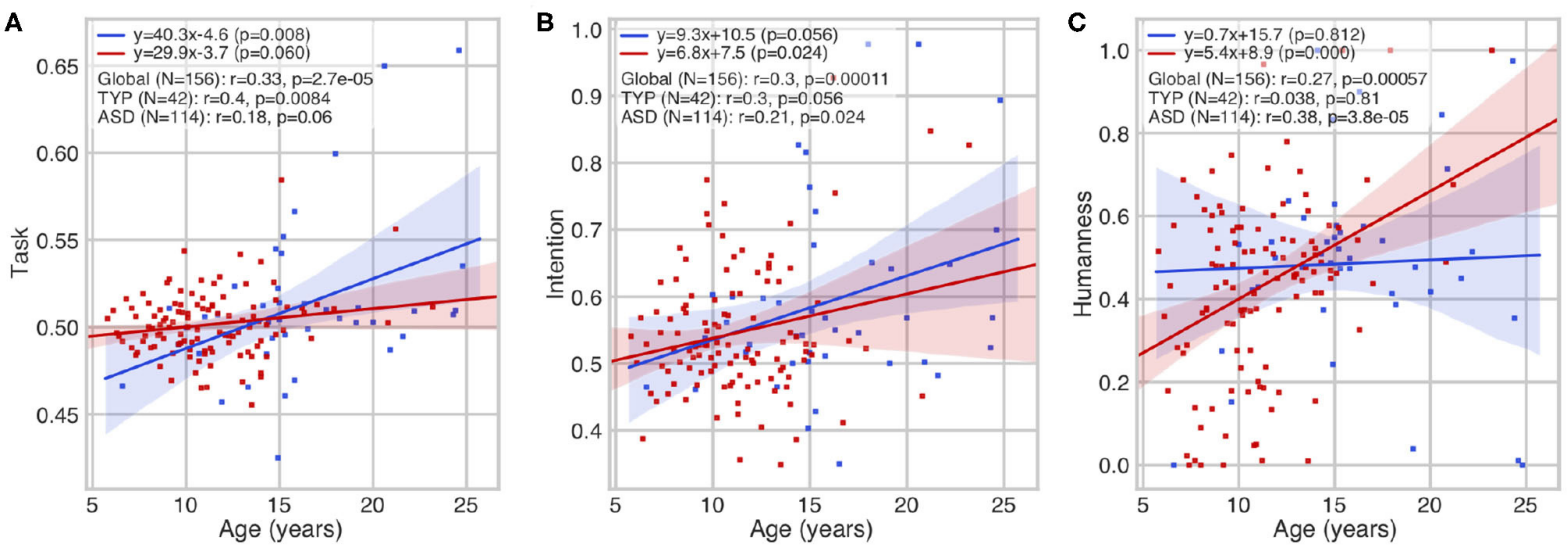
Developmental aspect of higher-level correlates of interpersonal synchrony (IS). Correlations between age at inclusion and task comprehension **(A)**, intention attribution **(B)**, and humanness **(C)** scores. The three scores show a remarkable positive correlation with age, suggesting a developmental trajectory of sociocognitive skills. No outliers were removed.

Within the entire cohort (both groups of participants with ASD and with typical development), a developmental trajectory was found with a statistically significant correlation of age with task comprehension (*r* = 0.33; *p* = 2.7e-05) (Figure 2A), intention attribution (*r* = 0.30; *p* = 0.00011) (Figure 2B), and humanness (*r* = 0.27; *p* = 0.00057) (Figure 2C). Only a few children with ASD diagnosis answered systematically the same rating of humanness across the whole experiment (*N* = 3 always human, i.e., humanness score = 1; *N* = 3 always robot, i.e., humanness score = 0). A significant interaction was observed between chronological age and comprehension score only in the control group (*r* = 0.40; *p* = 0.0084) (Figure 2A) (the older the participant is, the better the skills are) and with intention attribution (*r* = 0.21; *p* = 0.024) and humanness (*r* = 0.38; *p* = 3.8e-0.5) in the group with ASD.

### Comparison With Standardized Tests Using Normative Models

Using normative modeling allows us to correct any developmental bias on the HDC scores. We were then able to observe how these “age-controlled HDC scores” related to standard neuropsychological tests (Table 2).

**Table 2:** Summary of the main correlations between HDC scores and those from the NEPSY-II [affect recognition (AF) and theory of mind (TOM) subdomains], the Social Responsiveness Scale—second edition (SRS-2), and the Purdue Pegboard.

	**Motor (NM)**	**Coordination (NM)**	**Task (NM)**	**Intention (NM)**	**Humanness (NM)**
SRS-2	*r* = −0.22; *p* = 0.01^*^	*r* = 0.0031; *p* = 0.97	*r* = −0.22; *p* = 0.0086	*r* = −0.15; *p* = 0.076	*r* = −0.045; *p* = 0.6
NEPSY-II TOM	*r* = −0.081; *p* = 0.59	*r* = −0.35; *p* = 0.016^*^	*r* = −0.26; *p* = 0.08	*r* = 0.17; *p* = 0.24	*r* = −0.09; *p* = 0.55
NEPSY-II AF	*r* = 0.2; *p* = 0.18	*r* = −0.043; *p* = 0.78	*r* = 0.33; *p* = 0.023^*^	*r* = 0.16; *p* = 0.3	*r* = 0.11; *p* = 0.48
Purdue Pegboard	*r* = 0.14; *p* = 0.31	*r* = −0.2; *p* = 0.16	*r* = −0.015; *p* = 0.91	*r* = −0.25; *p* = 0.07	*r* = 0.13; *p* = 0.34

* *p < 0.05*.

**Figure 3:**
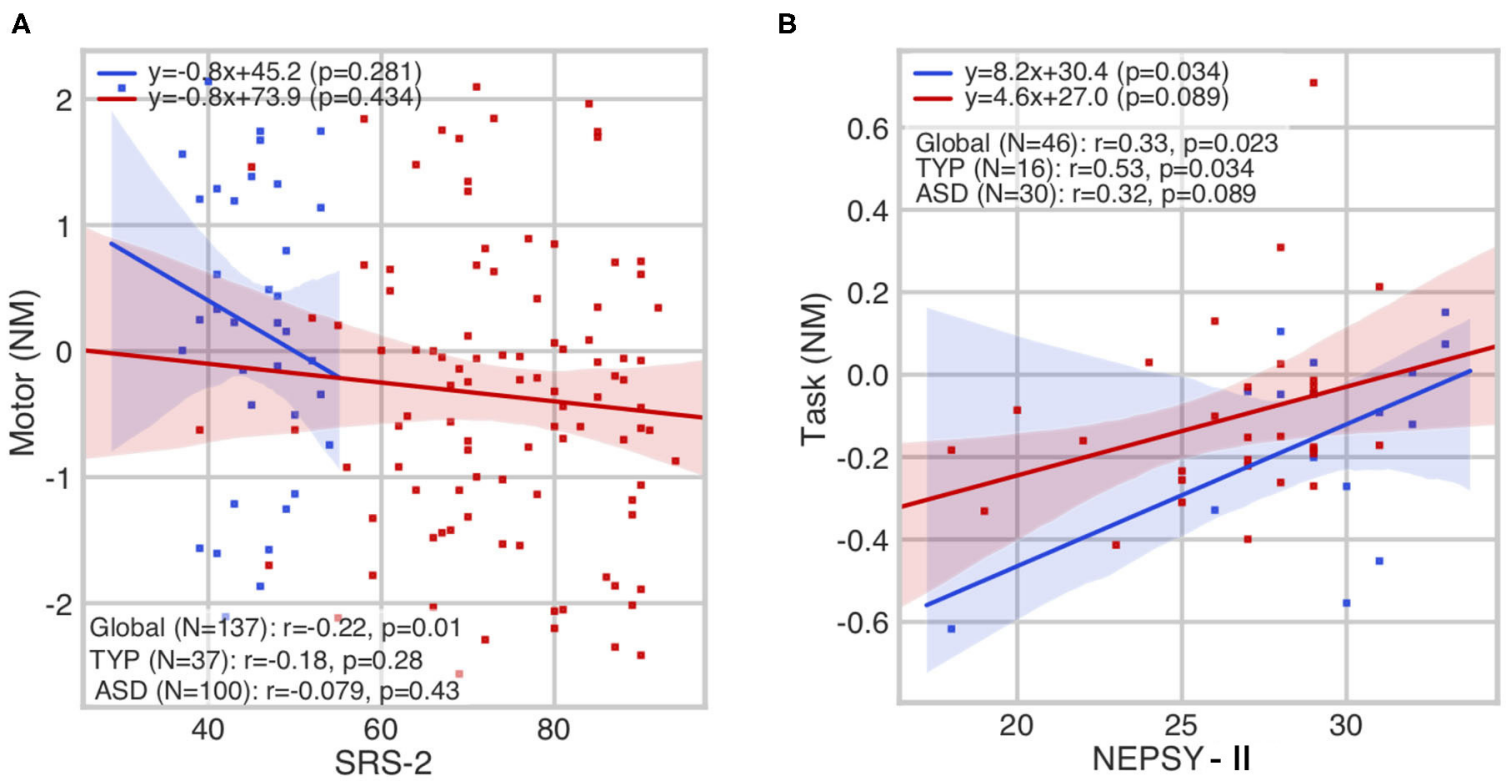
Significant correlations between sociocognitive and motor skills in children with (in red) autism spectrum disorder (ASD) or with typical development (TYP) (in blue): **(A)** SRS-2 vs. motor score: a dimensional diagnosis of ASD correlates with lower levels of motor skills; and **(B)** NEPSY-II affect recognition (AF) vs. HDC task score: greater cognitive abilities correlate with higher levels of affect recognition skill; NM, normative models.

We observed a significant interaction effect between the SRS-2 and motor score (*r* = −0.22; *p* = 0.01) (Figure 3A); high SRS scores (in favor of the diagnosis of ASD) are correlated with low motor scores. The NEPSY-II test showed a significant interaction effect between AF score and the HDC task comprehension score (*r* = 0.33; *p* = 0.02) (Figure 3B); good skills in the AF task of the NEPSY-II are associated with good scores at the HDC task comprehension score. The Supplementary Data Sheet contain the details of the correlation per group, along with the HDC scores before normative modeling correction.

### Global Comparative Analysis Between Participants With ASD and Typical Development Groups Using Normative Models

**Figure 4:**
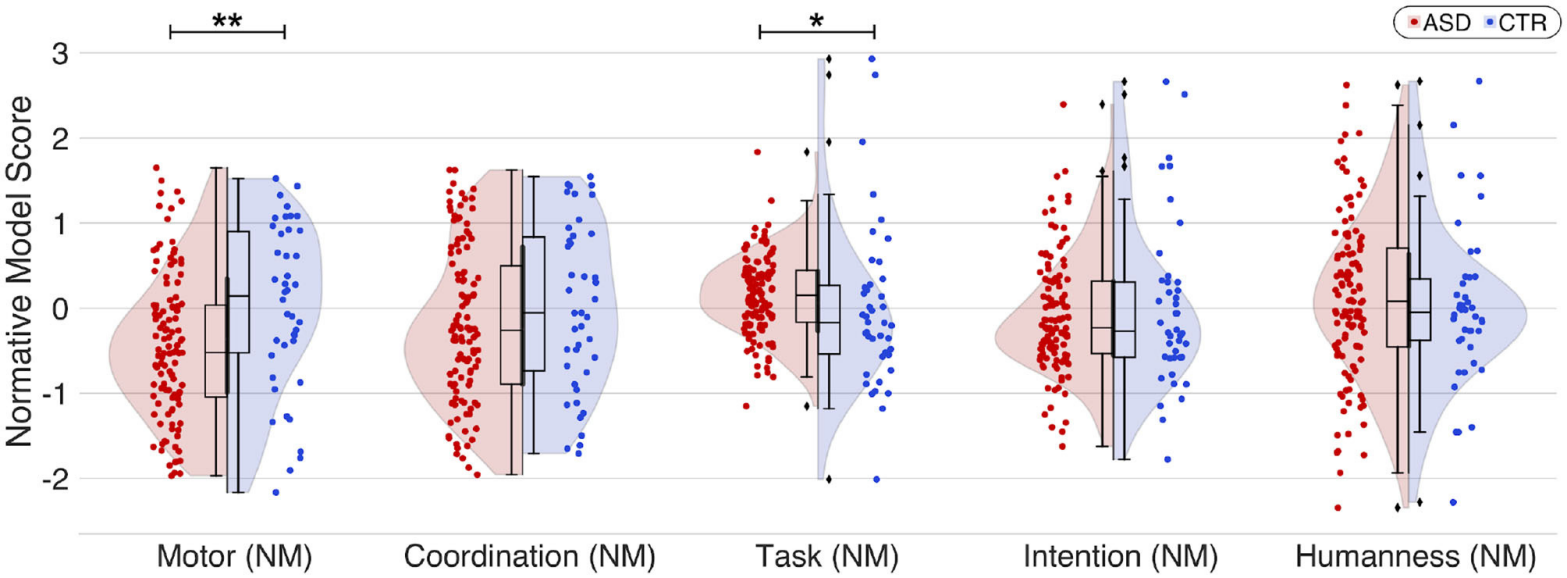
Comparison between the two groups with ASD and typical development for different behavioral scores derived from the HDC protocol and corrected with normative modeling (NM). Only the motor score really discriminates between the two populations (*d* = −0.5; *p* < 0.005^**^), with significantly lower results among ASD. The lines represent linear regressions. Colored areas: 95% confidence intervals (CI). Neurotypical participants: blue; participants with ASD: red. ^*^p < 0.05; ^**^p < 0.005.

Comparative analysis between the two groups revealed a statistically significant decrease of the motor score (*d* = −0.5; *p* = 0.0029) in individuals with an ASD diagnosis compared with individuals with typical development. We also observed evidence of better understanding of the task among participants with ASD diagnosis compared with those with typical development (*d* = 0.23; *p* = 0.0077). Interactions between the two groups for the other scores (coordination: *d* = −0.21, *p* = 0.12; intention: *d* = −0.12, *p* = 0.49; humanness: *d* = 0.12, *p* = 0.19) were not significant (Figure 4).

### HDC Scores Analysis by Subconditions

**Figure 5:**
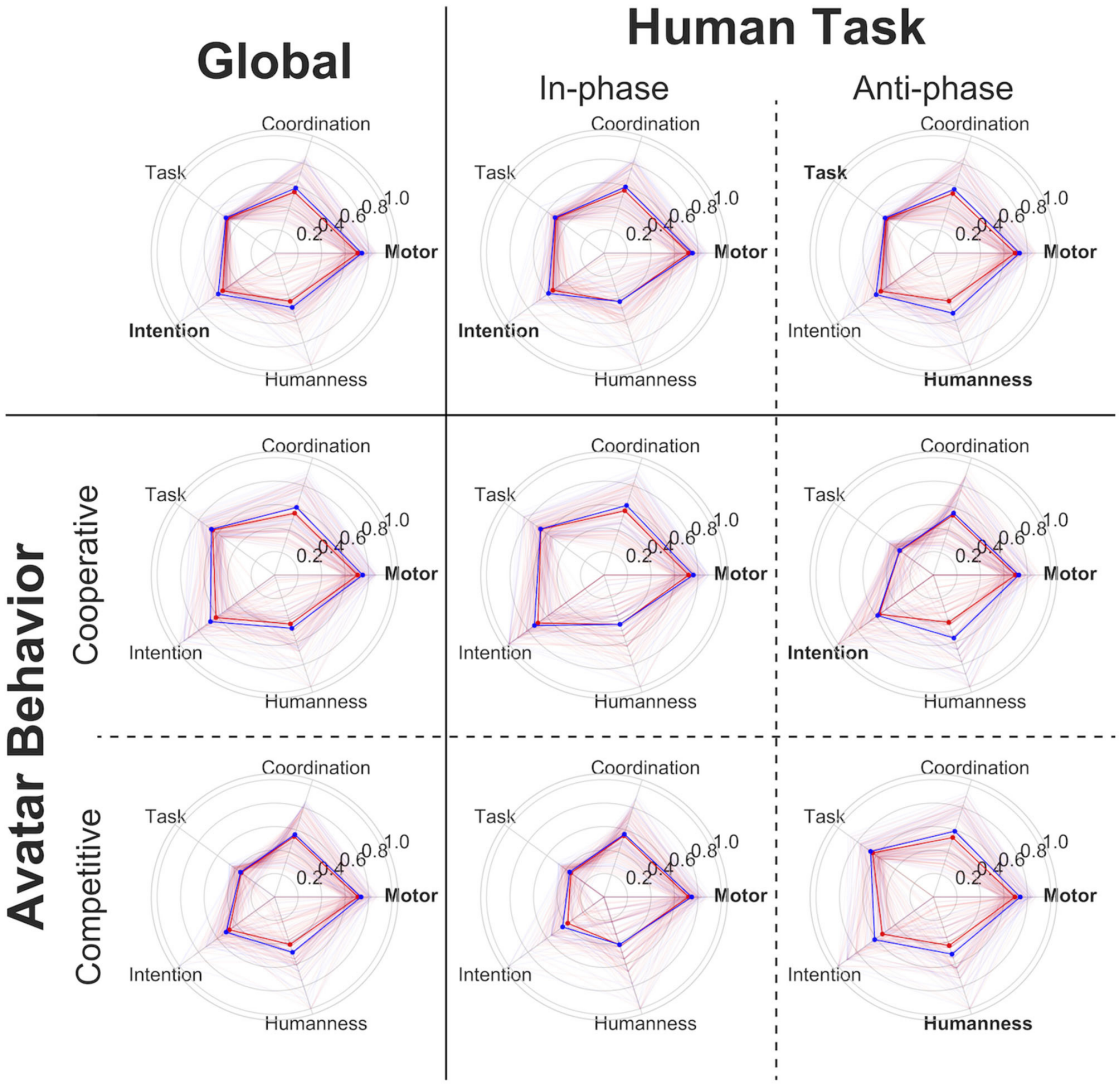
Score analysis by subcondition of the two groups (human task: in-phase/anti-phase and avatar behavior: cooperation/competition). Both the global and the human task were significant predictors of the motor score, with the control group having greater scores (coeff = 0.4413, *p* < 0.001^**^) and “in-phase” task also leading to higher scores (coeff = 0.2726, *p* = 0.016^*^). Controls are in blue and participants with ASD in red. Scores with statistically significant differences between participants with ASD and participants with typical development are in bold typeface. ^*^p < 0.05; ^**^p < 0.005.

Different subconditions are associated with the HDC paradigm: the diagnosis, the age, the avatar behavior, and the humanness or robotic character of the HDC (see Figure 5 for a summary). Multiple regression was thus calculated to predict the different normalized HDC scores based on the diagnosis (coded as 0 = ASD and 1 = CTR), age (in years), avatar behavior (coded as 0 = competitive and 1 = cooperative), and the humanness or robotic character discrimination task (coded as 0 = anti-phase and 1 = in-phase).

We found a significant regression equation for the motor score [*F*_(5, 618)_ = 7.634, *p* = 5.64e-07]. Both the diagnostic and the human task were significant predictors of the motor score, with the control group having higher scores (coeff = 0.44, *p* < 0.001), as well as the in-phase task (coeff = 0.27, *p* = 0.016). There was also a significant regression equation for the coordination score [*F*_(5, 618)_ = 3.252, *p* = 0.006], with age, as might be expected, a significant predictor (coeff = 0.0272, *p* = 0.02). A significant regression equation for task score [*F*_(5, 618)_ = 409.1, *p* = 2.42e-193] revealed that avatar behavior was a significant predictor, with a cooperative behavior of the VP having a huge effect on the task comprehension of participants (coeff = 6.96, *p* < 0.001). Further analysis of a significant regression equation for the intention score [*F*_(5, 618)_ = 28.84, *p* = 2.46e-26] showed that both human task and avatar behavior were significant predictors of VP intention. Participants tended to better detect the intention of the VP while “anti-phase” (coeff = −1.27, *p* < 0.001), and “in-phase” if the VP takes on a cooperative behavior (coeff = 2.31, *p* < 0.001).

For the motor score, detailed analysis indicates that during both VP “cooperative” (“in-phase”: *d* = −0.51; *p* = 0.006 and “anti-phase”: *d* = −0.59; *p* = 0.002) and “competitive” behavior (“in-phase”: *d* = −0.34; *p* = 0.025 and “anti-phase”: *d* = −0.35; *p* = 0.025), the task allows to distinguish the two groups (cf. Table 3).

**Table 3:** HDC score analysis by subconditions.

	**Motor (NM)**		**Coordination (NM)**		**Task (NM)**		**Intention (NM)**		**Humanness (NM)**	
	**Coop**	**Comp**	**Coop**	**Comp**	**Coop**	**Comp**	**Coop**	**Comp**	**Coop**	**Comp**
In-phase	*d* = −0.51 (*p* = 0.0059^*^)	*d* = −0.34 (*p* = 0.025*)	*d* = −0.26 (*p* = 0.098)	*d* = −0.087 (*p* = 0.35)	*d* = 0.12 (*p* = 0.17)	*d* = 0.067 (*p* = 0.47)	*d* = −0.018 (*p* = 0.47)	*d* = −0.084 (*p* = 0.35)	*d* = 0.34 (*p* = 0.014^*^)	*d* = 0.3 (*p* = 0.046^*^)
Anti-phase	*d* = −0.59 (*p* = 0.0015^**^)	*d* = −0.35 (*p* = 0.025^*^)	*d* = −0.3 (*p* = 0.054)	*d* = −0.11 (*p* = 0.29)	*d* = −0.067 (*p* = 0.41)	*d* = 0.16 (*p* = 0.23)	*d* = −0.2 (*p* = 0.14)	*d* = 0.067 (*p* = 0.31)	*d* = 0.012 (*p* = 0.49)	*d* = −0.25 (*p* = 0.058)

*Coop, cooperative behavior of the VP, Comp, competitive behavior of the VP, d, Cohen's d, p, p value*,

* *p < 0.05*,

** *p < 0.005*.

## Discussion

### Aim of the Study

The main objective of the study was to identify which HDC scores distinguish our two populations of children with and without a diagnosis of ASD and, thus, provide potential predictors of the condition. A particularly interesting aspect is that our results show the motor score discriminates between the two groups. Motor abnormalities in the disorder are widely described. However, they are still currently little taken into account in the diagnosis. As a reminder, the HDC is validated in adults as an instrument to artificially recreate a social interaction, from low-level (motor and coordination scores) to higher-level domains of social coordination [intention attribution to another and human (/or robotic) judgment of an interaction]. Our secondary objective was to study the developmental trajectory of HDC scores and to demonstrate by a valid scientific approach that interpersonal synchrony captures the coupling between low-level sensorimotor and high-level sociocognitive skills in a population of children.

### Developmental Aspects of Sociocognitive Skills and Intervention Based on Interpersonal Synchrony in Children With ASD

The literature on interpersonal synchrony attests to the significance of development and plasticity in affording therapeutic detection and action (39, 41, 43). The present HDC results are in line with this developmental aspect of sociocognitive skills, with significant effects of intention attribution, humanness, and task comprehension in children and adolescents with and without ASD. Interventions targeting early development of socially synchronous interactions in toddlers with ASD attest to its effectiveness (39), with improvement in child language comprehension being linked to the severity of ASD symptoms (63). The neurodevelopmental trajectory observed here only in the group with ASD is fully in line with this picture.

### Coupling Between Low-Level Sensorimotor and High-Level Sociocognitive Skills

The present findings also show that affect recognition may be associated with better task comprehension. Greater cognitive abilities are correlated with a higher level of affect recognition skill hinting at the possibility of a mediating effect of IQ on the recognition of emotions. At the same time, we found that lower motor skills are associated with a higher probability of a dimensional diagnosis of ASD. Motricity in ASD will be discussed further, but this result suggests a linkage between the so-called “lower-level” motor skills and “higher-level” social–cognitive skills in this population. Some support already exists for a strong pairing between the mirror and mentalizing systems during communicative gestures, suggesting a cognitive–motor coupling in children (64). The mechanisms involved range from the release of endogenous opioids (dopamine, endorphins, serotonin, and oxytocin) (65, 66) to the recruitment of now well-described neural processes (67, 68). From an evolutionary perspective, IS is thought to play a role in shared common goals that lead to: a) cooperative expectations and joint action behaviors (69); b) shared basic affective states and emotions; c) better attribution ability of one's self and others; and d) in general, better comprehension of social situations (70).

### Motor Skills as a Developmental Marker of Children and Adolescents at Risk With ASD

The motor score is the only HDC measure that allows a distinction between the two groups. In overall terms, this motor low-level score is found to be statistically lower among participants with ASD, confirming current data finding altered motor skills in ASD. Despite the small sample size, it is interesting to note that the HDC motor score is also one of the two scores (together with the motor coordination score) on which the classification into two clusters is essentially based—a classification that significantly respects the status of participants (see Supplementary Material) (71). These results demonstrate the essential nature of motor assessment, including the use of HDC, in participants with ASD, suggesting a major role in ASD diagnosis (72). Alterations in motor control (38), and particularly of executive motor control (73), have been widely demonstrated in children with ASD. However, although motor disorders are associated with the diagnosis of children with ASD in 50 to 80% (74), their prevalence apparently increasing with age (75), they remain underdiagnosed in clinical practice (1.34%) (75). On the other hand, the estimated prevalence of motor disorders (36%) makes them almost as frequent as cognitive disorders (38%) among children under 6 years (75). Children and adolescents with ASD tend to have difficulties in planning and sequencing movements (76), which are also associated with higher levels of neuromotor noise (77) [i.e., disturbing action (motor commands) and perception (sensory feedback) (20)]. Such variability can have multiple substrates but relies on hypotheses that can be explained using Bayesian models, namely an imbalance between prediction, inputs, and expectations (78). Indeed, ASD is associated with alterations in the ability to integrate social stimuli (79) and a reduced ability to incorporate somatosensory and visual information into accurate motor responses (37). Moreover, some studies now describe deficits in joint-attention as an endophenotype of ASD (80, 81). Vis-à-vis our results, children with ASD may have difficulties in sustaining attention long enough to perceive the stimulus. Further analysis showed that the instruction given to the participant (human task: “in-phase”) is associated with a better motor score among the control group (coeff = 0.2726, *p* = 0.016^*^). Also, we observed interactions by subcondition. Children with ASD tend to have lower results than children with typical development in all the conditions, i.e., “in-phase” as well as “anti-phase” during both competitive and cooperative behavior of the avatar (Table 3). This result accords with Wang et al. (82) who reported that during a cooperative task of synchronization (i.e., “in-phase”), children with severe diagnosis of ASD tend to exhibit lower neural activity.

### Sociocognitive Skills Based on Interpersonal Synchrony

Mentalizing deficits have repeatedly been described in the population with ASD (83). Mentalizing requires preserved metacognitive skills, yet metacognitive monitoring is found diminished in children with ASD (84). Higher-level scores (intention attribution and task comprehension) demand efficient use of metacognitive processes. However, for both scores, we did not observe a group effect: it seemed easier for participants to detect the intention of the VP when it takes on a cooperative behavior whether “anti-phase” or “in-phase”. Furthermore, comprehension of the task is better if the avatar is cooperative. A result that seems difficult to interpret is that participants with an ASD diagnosis appear to have a better understanding of the task than controls. This could be due to the tendency of ASD children to generate mainly the same movement “in-phase” with the avatar without taking the instruction into account–or it could be due to the lack of a real-time social context (85). Such a possibility of bias may produce a false positive result.

In addition, only children with a diagnosis of ASD showed a persistence of the same response in the assessment of the attribution of humanness (or robotic) judgment to the avatar. This result reinforces the previous observation of an insistence on sameness in ASD (86, 87) and may be consistent with the repetitive behaviors that are part of the diagnosis (DSM-5).

### Implications of Findings for Clinical Practice and Public Health

One of the challenges of the present approach is to develop an application of HDC that can be used for the early assessment and training of motor coordination and interpersonal synchrony in order to improve social skills (42). The aim of our study was also to offer standardized ways to assess the efficacy of the HDC. We were able to generate percentile ranks for each HDC score from the results obtained in control patients (see Supplementary Table 1). This step made it possible to estimate a child's skills for each assessment.

Research has tried to identify clinical markers in ASD ranging from early signs of regression patterns (44, 88) to atypical neural responses of gaze (89). Later possibilities include neurological soft signs (90), abnormalities of sensorimotor priors (34), and anomalies in proprioceptive and sensory motor development (including alteration of motor priors, micromovements, and the presence of noise in sensory motor variables that may be associated with lack of embodiment) (34).

Systematic and reliable metrics of the HDC, normalized across developmental trajectories by means of normative models (91), could help predict phenotypic profiles and, thus, refine the diagnosis, associated comorbidities, and stratification of the disorder. Here, we highlighted how HDC measures can provide new markers of ASD, either alone or in combination with psychometric scales for assessing social and motor coordination skills. Despite the limited sample size, exploratory analyses of stratification and multivariate predictive diagnosis (see Supplementary Material for more details on the methodology and the preliminary results) tend to confirm the potential of the HDC paradigm for ASD diagnostics with the motor and coordination scores, individually, and/or combined with other clinical evidence of ASD, appearing as the most promising candidates. Clearly, there is a need to develop dedicated HDC-based predictive models and for further data collection and analyses to be carried out to assess them rigorously (92). Many studies have reported divergences in the core symptoms of ASD by gender (93) and level of intellectual disability (94). Future studies in larger cohorts will allow disentangling such key factors in the development of interpersonal synchrony.

## Conclusion

The HDC is an effective means to evaluate interpersonal synchrony at both low and high levels of social cognition during live interactions. It can also probe the developmental aspects of their evolving relationship. On the other hand, the psychometric evaluation of HDC provides reliable, reproducible, objective, and standardized scores, derived from a natural movement. As a new psychometric test, HDC provides motor and social markers that help to improve the early detection of neurobehavioral abnormalities during human interaction. The HDC paradigm also provides a dynamical basis for the development of further therapeutic approaches, for instance in the area of serious games (e.g., in mixed reality: https://vimeo.com/277085489).

## Data Availability Statement

The datasets generated for this study will not be made publicly available because of the clauses in the ethical consents.

## Ethics Statement

The studies involving human participants were reviewed and approved by Hospital Robert Debré. Written informed consent to participate in this study was provided by the participants' legal guardian/next of kin.

## Author Contributions

FB, AL, and AP collected all the data. FB, AL, FA, and AM worked on the inclusion of patients and their clinical exploration. FB, AL, YB, and DE participated in both analysis and writing. RD and TB participated in the design and relecture. GD participated in the design, analysis, and writing. All authors contributed to the article and approved the submitted version.

## Conflict of Interest

The authors declare that the research was conducted in the absence of any commercial or financial relationships that could be construed as a potential conflict of interest.

## Supplementary Material

Supplementary Data Sheet Additional methods regarding the calculation of the HDC scores, the stratification using clustering analysis, and the supervised learning with logistic regression.

Supplementary Figure 1 Stratification based on HDC scores. **(A)** Optimal number of clusters between k = 2 to k = 20 obtained by NbClust with 26 indices. The majority vote (9 of 26) indicates k = 2 as the optimal cluster configuration. **(B)** Corresponding Hierarchical Clustering using Euclidian distance and the Ward method.

Supplementary Table 1 HDC percentile ranks by age group.

Supplementary Table 2 Comparison of HDC and clinical scores between the two clusters identified.
