# Supplementary material for: Interactive Psychometrics for Autism With the Human Dynamic Clamp: Interpersonal Synchrony From Sensorimotor to Sociocognitive Domains
Source: Front Psychiatry. 2020 Nov 26;11:510366. doi: 10.3389/fpsyt.2020.510366 (PMC7725713; doi:10.3389/fpsyt.2020.510366)
Supplement: Supplementary Data Sheet — Additional methods regarding the calculation of the HDC scores, the stratification using clustering analysis, and the supervised learning with logistic regression. [file Data_Sheet_1.docx]

# *SUPPLEMENTARY MATERIAL*

***1. HDC scores***

**Motor score**: measures the difference of amplitude of imitative gestures between the participant and the VP. A mean is obtained for each trial.

$Motor = \frac{1}{1+\left( \frac{{Amp}_{human}-{Amp}_{avatar}}{{Amp}_{human}+{Amp}_{avatar}} \right)^{2}}$ (Equ. 1)

where ${Amp}_{human}$and ${Amp}_{avatar}$ are respectively the amplitude of the movement of the participant and the VP, both extracted from a Hilbert transform. The latter was used to extract phase and amplitude of the movements, so they can respectively help in quantifying morphological and temporal similarity between the behavior of the participant and the avatar.

**Coordination score**: corresponds to the temporal index of imitation. This measure is based on the frequency parameter and evaluates the shift of frequency between the avatar and the human participant. A mean is obtained for each trial.

$Coordination = \frac{1}{N}\sum_{t=0}^{N} \left| e^{i\left( \varphi_{human}-\varphi_{avatar} \right)} \right|$ (Equ. 2)

where $\varphi_{human}$ and $\varphi_{avatar}$ are respectively the phase of the movement of the participant and the VP, both extracted from a Hilbert transform, $\left| \right|$is the norm in the complex plane, and $N$is the total number of samples;

**Task score:** based on the ongoing relative phase of the VP and the participant, taking into account the task condition.

$Task =1-\frac{1}{N}\sum_{t=0}^{N} \frac{arg\left( e^{i\left( \phi-\theta\right)} \right)}{\pi}$ (Equ. 3)

where $\phi$is the relative phase, *i.e.* the difference between the phase of the movement of the participant and the VP, $\theta$is the goal of the task, i.e. 0 if in-phase and $\pi$if anti-phase, N is the total number of samples, and arg() indicates the mathematical function returning the angle in radians of a complex number;

**Intention score:** evaluates the ability of the participant to properly attribute intention toward the "cooperative" or "competitive" behavior of the VP.

$Intention=1-\left| {Behavior}_{avatar}-CoopReport_{human} \right|$ (Equ. 4)

where ${Behavior}_{avatar}$is 0 when the VP is competitive *i.e*. conflicting goal with the participant, and 1 when it is cooperative (i.e. shared goal with the human participant. $CoopReport_{human}$ is the rating of cooperativeness of the avatar reported by the human participant at the end of each trial;

**Humanness score:** reflects quantitatively the impression of the participant on the human or robotic character of the VP.

$Humanness=1-\left| {Identity}_{avatar}-HumReport_{human} \right|$ (Equ. 5)

where ${Identity}_{avatar}$is 0 when the VP is a robot and 1 when it is a human, and $HumReport_{human}$ is the rating of the humanness of the VP reported by the participant at the end of each trial.

**2. Stratification using clustering analysis**

We used hierarchical clustering to simultaneously test how HDC behavioral measures taken collectively could serve as a proxy for diagnostic and stratification biomarkers of ASD. This procedure divides participants into clusters so that within these clusters the participants are more similar than those of other clusters. We used the NbClust R package (Charrad, Ghazzali, Boiteau, and Niknafs, 2014), to estimate in an objective, data-driven fashion the optimal number of clusters. A “majority vote” across 26 indices determines the “best” number of clusters k in the data given a specific linkage method. Here we used the Ward's method on the normalized HDC scores and search possible solutions from k=2 to k=20. Analyses on the 156 participants including 114 participants with ASD give a majority vote (9 of 26) for a 2-cluster solution (Supplementary Figures 1 & 2).

We validate internally the clusters with T-test and Cohen d on the HDC measures and externally with Chi2 test on the diagnostic status, and T-test and Cohen d on the NEPSY and Purdue Pegboard scores. Supplementary Table 2 shows a two-group t-test for each of the 8 factor variables between clusters 1 and 2. Of the 8 factor variables, 4 achieved significant difference (all HDC scores, in order of significance value): task score (d = 0.44, p = 0.02), Intention score (d = 0.03, p = 0.03), motor score (d = 2.28, p = 5 x 10^-26^), and coordination score (d = 2.058, p = 2 x 10^-24^). No significant results were found between the two clusters for the NEPSY scores and the Purdue Pegboard scores. We also analyzed the repartition within the two clusters of the population by considering the diagnosis with a significant difference (Pearson Chi^2^ = 3.79, p = 0.05. Taken together, these results support clustering based on HDC, especially with Motor and Coordination scores and assigning subjects according to their diagnostic status.

**3. Supervised learning with logistic regression**

In parallel, we also used logistic regression to predict diagnosis using the inverse logistic function of penalized linear combinations of the HDC dimensions on the full dataset (n=156, ASD=114). Logistic regression is a simple linear classification method with limited flexibility at the same time as helping to avoid overfitting, in line with best-practice recommendations (Dadi et al. 2019). As the right penalty term C (a Gaussian shrinkage prior) is rarely known in advance, we selected this hyper-parameter with 3-fold nested cross-validation considering 50 candidate values from the equally spaced logarithmic grid between 1e-4 and 1e4. A nested standard scaler was applied. All computation was carried out using the Python library scikit-learn (Pedregosa et al. 2011) using Python 3.7. We estimated generalization performance with 10-fold stratified cross-validation at an average AUC score of around 0.63 (+/- 0.19). This performance was considerably variable, with its average score unlikely under the Null-hypothesis according to 1000 permutations of the diagnostic labels (p < 0.024). This result suggests that the HDC contains generalizable pieces of information that may complement other clinical or neuroscientific assessments.

In a second step, we examined the reduced subset with complete clinical information using the identical model configuration. For the HDC, we estimated a generalization performance of AUC = 0.81 (+/-0.19). For NEPSY and the Purdue Pegboard combined, we estimated a performance of AUC = 0.75 (+/-0.3), and for a model based on the entire data set, a generalization performance of AUC = 0.83 (+/-0.33). Although these preliminary results are in line with the view that the HDC contains unique information, it is important to note the performance difference between the HDC on the full data (n=156) and the HDC on the reduced subset (n=42). Higher performance on the reduced data set is suggestive of sampling bias and overfitting. When the dataset is too small, neither cross-validation nor shrinkage priors can guard against unstable parameter estimates and erroneous predictions (Poldrack, Huckins and Varoquaux, 2019).

**References**

Charrad, M., Ghazzali, N., Boiteau, V., Niknafs, A., & Charrad, M. M. (2014). Package ‘nbclust’. Journal of statistical software, 61, 1-36.

Dadi, K., Rahim, M., Abraham, A., Chyzhyk, D., Milham, M., Thirion, B., ... & Alzheimer's Disease Neuroimaging Initiative. (2019). Benchmarking functional connectome-based predictive models for resting-state fMRI. *Neuroimage*, *192*, 115-134.

Pedregosa, F., Varoquaux, G., Gramfort, A., Michel, V., Thirion, B., Grisel, O., ... & Vanderplas, J. (2011). Scikit-learn: Machine learning in Python. *the Journal of machine Learning research*, *12*, 2825-2830.

Poldrack, R. A., Huckins, G., & Varoquaux, G. (2020). Establishment of best practices for evidence for prediction: a review. *JAMA psychiatry*, *77*(5), 534-540.

**
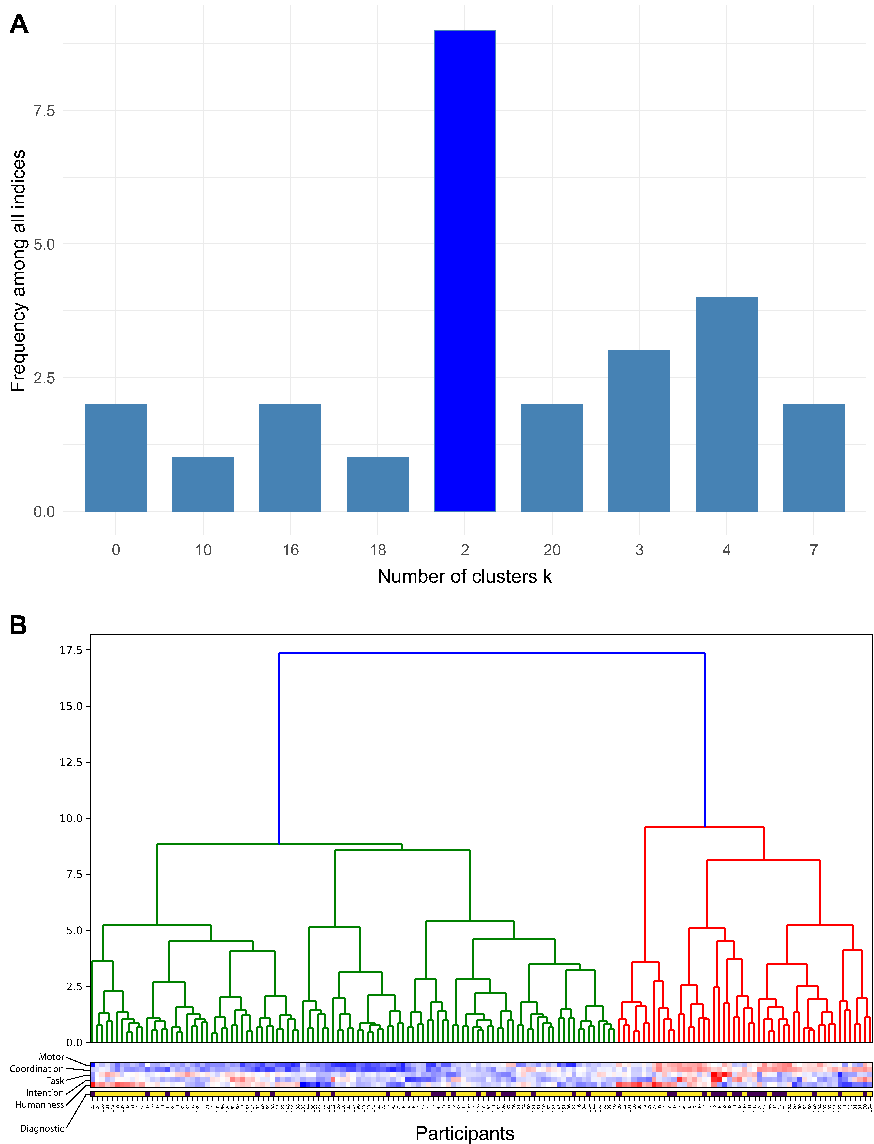
**

**Supplementary Figure 1.** Stratification based on HDC scores. A) Optimal number of clusters between k=2 to k=20 obtained by NbClust with 26 indices. The majority vote (9 of 26) indicates k=2 as the optimal cluster configuration. B) Corresponding Hierarchical Clustering using Euclidian distance and the Ward method.

***Supplementary Table 1***

*HDC percentile ranks by age group.*

| **Age** | 5-10 | | | 10-13 | | | 13-15 | | | 15-26 | | |
| --- | --- | --- | --- | --- | --- | --- | --- | --- | --- | --- | --- | --- |
| **Percentiles** | 5e | 50e | 95e | 5e | 50e | 95e | 5e | 50e | 95e | 5e | 50e | 95e |
| **Motor** | 0.65 | 0.72 | 0.86 | 0.63 | 0.70 | 0.81 | 0.62 | 0.79 | 0.88 | 0.58 | 0.73 | 0.88 |
| **Coordination** | 0.24 | 0.55 | 0.85 | 0.24 | 0.40 | 0.83 | 0.32 | 0.63 | 0.90 | 0.325 | 0.54 | 0.89 |
| **Task** | 0.23 | 0.48 | 0.80 | 0.27 | 0.50 | 0.75 | 0.19 | 0.49 | 0.79 | 0.24 | 0.50 | 0.84 |
| **Intention** | 0.26 | 0.51 | 0.69 | 0.34 | 0.56 | 0.87 | 0.0 | 0.61 | 1.0 | 0.18 | 0.58 | 1.0 |
| **Humanness** | 0.0 | 0.17 | 0.30 | 0.27 | 0.50 | 0.76 | 0.07 | 0.55 | 1.00 | 0.0 | 0.50 | 0.95 |

**Supplementary Table 2**

*Comparison of HDC and clinical scores between the two clusters identified.*

|  | **Motor (NM)** | **Coordination (NM)** | **Task (NM)** | **Intention (NM)** | **Humanness (NM)** | **Purdue Pegboard** | **NEPSY Social Perception** | **NEPSY Affect Recognition** |
| --- | --- | --- | --- | --- | --- | --- | --- | --- |
| **Comparison of variables between the two clusters** | d=2.28  (p=5.4 x 10^-26^**) | d=2.05  (p=2.16 x 10^-24^**) | d=0.44  (p=0.02*) | d=0.43 (p=0.03*) | d=0.31 (p=0.10) | d=0.25 (p=0.38) | d=0.37 (p=0.24) | d=0.39 (p=0.21) |

d = cohen-d, * = p<0.05, ** = p<0.005.
