# Supplementary figures and images for: Interactive Psychometrics for Autism With the Human Dynamic Clamp: Interpersonal Synchrony From Sensorimotor to Sociocognitive Domains
Source: Front Psychiatry. 2020 Nov 26;11:510366. doi: 10.3389/fpsyt.2020.510366 (PMC7725713; doi:10.3389/fpsyt.2020.510366)

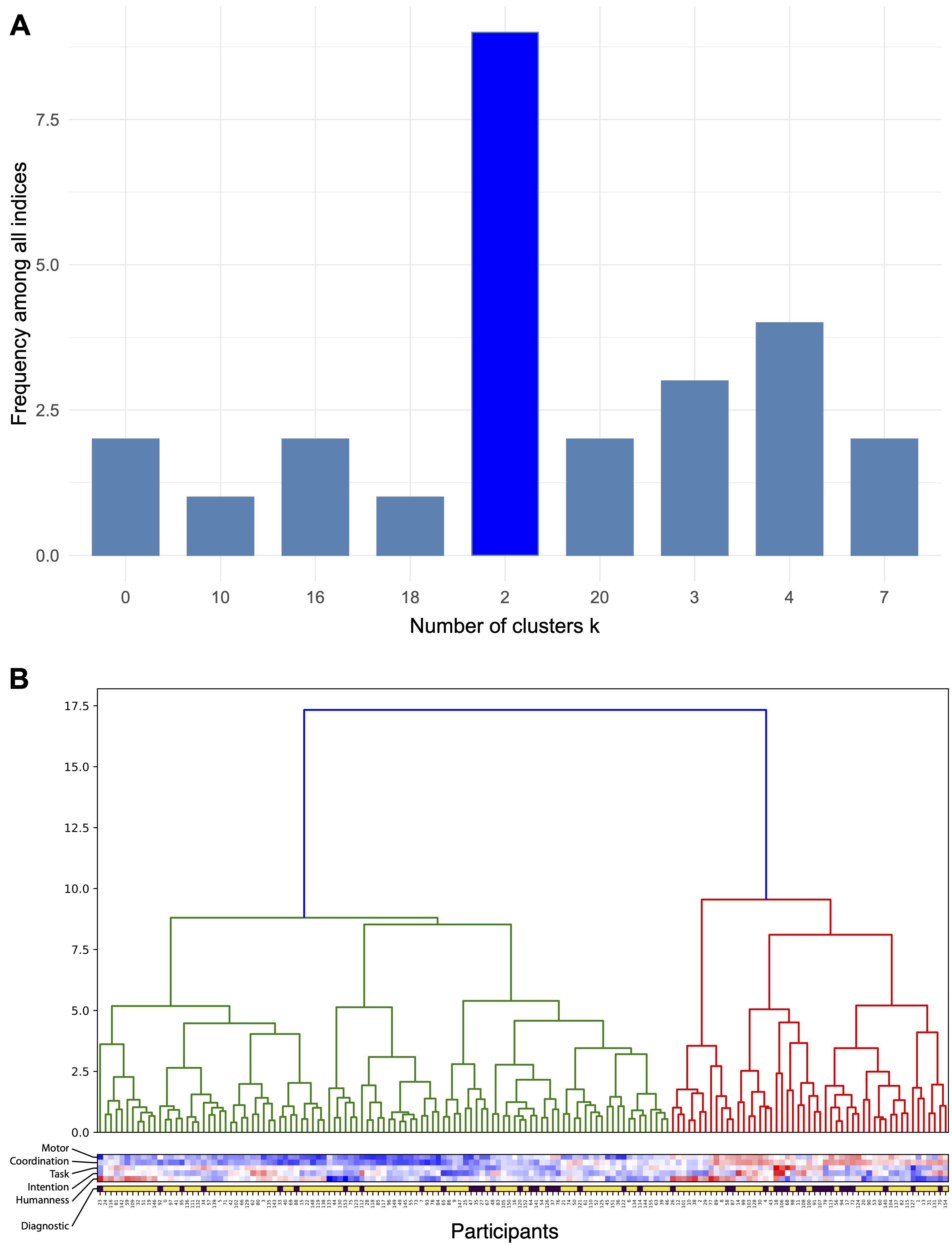

Supplement: Supplementary Figure 1 — Stratification based on HDC scores. (A) Optimal number of clusters between k = 2 to k = 20 obtained by NbClust with 26 indices. The majority vote (9 of 26) indicates k = 2 as the optimal cluster configuration. (B) Corresponding Hierarchical Clustering using Euclidian distance and the Ward method. [file Image_1.JPEG]
