# Supplementary material for: Interactive Psychometrics for Autism With the Human Dynamic Clamp: Interpersonal Synchrony From Sensorimotor to Sociocognitive Domains
Source: Front Psychiatry. 2020 Nov 26;11:510366. doi: 10.3389/fpsyt.2020.510366 (PMC7725713; doi:10.3389/fpsyt.2020.510366)
Supplement: Supplementary Table 1 — HDC percentile ranks by age group. [file Table_1.DOCX]

***Supplementary Table 1 :*** *HDC percentile ranks by age group.*

| *Age* | *5-10* | | | *10-13* | | | *13-15* | | | *15-26* | | |
| --- | --- | --- | --- | --- | --- | --- | --- | --- | --- | --- | --- | --- |
| *Percentiles* | *5e* | *50e* | *95e* | *5e* | *50e* | *95e* | *5e* | *50e* | *95e* | *5e* | *50e* | *95e* |
| *Motor* | *0.65* | *0.72* | *0.86* | *0.63* | *0.70* | *0.81* | *0.62* | *0.79* | *0.88* | *0.58* | *0.73* | *0.88* |
| *Coordination* | *0.24* | *0.55* | *0.85* | *0.24* | *0.40* | *0.83* | *0.32* | *0.63* | *0.90* | *0.325* | *0.54* | *0.89* |
| *Task* | *0.23* | *0.48* | *0.80* | *0.27* | *0.50* | *0.75* | *0.19* | *0.49* | *0.79* | *0.24* | *0.50* | *0.84* |
| *Intention* | *0.26* | *0.51* | *0.69* | *0.34* | *0.56* | *0.87* | *0.0* | *0.61* | *1.0* | *0.18* | *0.58* | *1.0* |
| *Humanness* | *0.0* | *0.17* | *0.30* | *0.27* | *0.50* | *0.76* | *0.07* | *0.55* | *1.00* | *0.0* | *0.50* | *0.95* |
