# Supplementary material for: Interactive Psychometrics for Autism With the Human Dynamic Clamp: Interpersonal Synchrony From Sensorimotor to Sociocognitive Domains
Source: Front Psychiatry. 2020 Nov 26;11:510366. doi: 10.3389/fpsyt.2020.510366 (PMC7725713; doi:10.3389/fpsyt.2020.510366)
Supplement: Supplementary Table 2 — Comparison of HDC and clinical scores between the two clusters identified. [file Table_2.DOCX]

***Supplementary Table 2 :*** *Comparison of HDC and clinical scores between the two clusters identified.*

|  | **Motor (NM)** | **Coordination (NM)** | **Task (NM)** | **Intention (NM)** | **Humanness (NM)** | **Purdue Pegboard** | **NEPSY Social Perception** | **NEPSY Affect Recognition** |
| --- | --- | --- | --- | --- | --- | --- | --- | --- |
| **Comparison of variables between the two clusters** | d=2.28  (p=5.4 x 10^-26^**) | d=2.05  (p=2.16 x 10^-24^**) | d=0.44  (p=0.02*) | d=0.43 (p=0.03*) | d=0.31 (p=0.10) | d=0.25 (p=0.38) | d=0.37 (p=0.24) | d=0.39 (p=0.21) |

d = cohen-d, * = p<0.05, ** = p<0.005.
